# Prenatal screening above aneuploidies that is Pre-Eclampsia

**DOI:** 10.1186/1755-8166-7-S1-O6

**Published:** 2014-01-21

**Authors:** Gurjit Kaur

**Affiliations:** 1 Department of Physiology, Consultant Incharge, Genetic Centre, GMCH-32, Chandigarh, India

## Background

Pre-Eclampsia is defined as high blood pressure and excess protein in the urine after 20 weeks of pregnancy in a normotensive woman. Even a slight increase in blood pressure may be a sign of preeclampsia. Despite extensive clinical trials no therapeutic approaches are available for either treatment or prevention of preeclampsia. Removal of placenta remains the only solution for resolution of preeclampsia which necessitates premature deliveries and can have adverse outcomes of low birth-weight babies, at the same time increasing the risk of maternal and neonatal mortality and morbidity worldwide. The concentrations of several placental proteins, inflammatory cytokines and growth factors are altered in the maternal circulation of women with preeclampsia. Nonetheless, early pregnancy screening for preeclampsia remains insufficient, and randomized controlled trials that used biomarkers to identify high risk women have been disappointing, perhaps because the sensitivity of most of these markers is high in the second or third trimester, long after the placental dysfunction is already established. At Genetic Centre, GMCH-32,Chandigarh we have tried to correlate first trimester biochemical and ultrasonographic markers (viz. Pregnancy Associated Plasma Protein-A, Placental Growth Factor, Doppler Pulsatility Index) with establishment of preeclampsia and other obstetrical complications (fetal growth restriction, pre-mature delivery, risk of miscarriage, still-birth) in pregnant women. This study will help in determining the possible diagnostic utility of PAPPA, PlGF, and Doppler Pulsatility Index as sensitive and specific biomarkers for screening early onset of pre-eclampsia.

## Materials and methods

All pregnant women visiting Government Medical College and Hospital, Chandigarh in first trimester of pregnancy are tested for PAPPA, PlGF, and Doppler Pulsatility Index after thorough counselling and written informed consent. PAPPA, PlGF and Doppler Pulsatility Index are quantitatively analyzed and multiple of medians are obtained of approximately 1000 pregnant ladies.

## Result and conclusion

In order to relate mode of delivery (NVD/LSCS) with PAPPA, PlGF, and Doppler Pulsatility Index an initial sample size of 222 was taken for analysis. The association of mode of delivery was carried out using chi square test and it has been observed that PAPPA risk has a significant association with mode of delivery however PlGF and Doppler Pulsatility Index do not have a significant association. However this is an ongoing study and statistical analysis of more samples is needed to get a significant association.

